# Concordance analysis of cerebrospinal fluid with the tumor tissue for integrated diagnosis in gliomas based on next-generation sequencing

**DOI:** 10.3389/pore.2023.1611391

**Published:** 2023-09-26

**Authors:** Qiang Wang, Qiujin Liang, Wuting Wei, Wenhao Niu, Chong Liang, Xiaoliang Wang, Xiaoxuan Wang, Hao Pan

**Affiliations:** ^1^ Department of Neurosurgery, Jinling Hospital, Nanjing, China; ^2^ State Key Laboratory of Neurology and Oncology Drug Development, Jiangsu Simcere Diagnostics Co., Ltd., Nanjing, China

**Keywords:** cerebrospinal fluid, glioma, circulating tumor DNA, integrated diagnosis, IDH1 R132C

## Abstract

**Purpose:** The driver mutations of gliomas have been identified in cerebrospinal fluid (CSF). Here we compared the concordance between CSF and tumor tissue for integrated diagnosis in gliomas using next-generation sequencing (NGS) to evaluate the feasibility of CSF detection in gliomas.

**Patients and methods:** 27 paired CSF/tumor tissues of glioma patients were sequenced by a customized gene panel based on NGS. All CSF samples were collected through lumbar puncture before surgery. Integrated diagnosis was made by analysis of histology and tumor DNA molecular pathology according to the 2021 WHO classification of the central nervous system tumors.

**Results:** A total of 24 patients had detectable circulating tumor DNA (ctDNA) and 22 had at least one somatic mutation or chromosome alteration in CSF. The ctDNA levels varied significantly across different ages, Ki-67 index, magnetic resonance imaging signal and glioma subtypes (*p* < 0.05). The concordance between integrated ctDNA diagnosis and the final diagnosis came up to 91.6% (Kappa, 0.800). We reclassified the clinical diagnosis of 3 patients based on the results of CSF ctDNA sequencing, and 4 patients were reassessed depending on tumor DNA. Interestingly, a rare *IDH1* R132C was identified in CSF ctDNA, but not in the corresponding tumor sample.

**Conclusion:** This study demonstrates a high concordance between integrated ctDNA diagnosis and the final diagnosis of gliomas, highlighting the practicability of NGS based detection of mutations of CSF in assisting integrated diagnosis of gliomas, especially glioblastoma.

## Introduction

Central nervous system (CNS) tumors present survival challenges for people because of high incidence and high mortality. Glioma is the most common primary CNS tumor, accounting for 80.8% of the malignant CNS tumors in adults [1]. Integrated diagnosis of the CNS tumors has been updated and highlighted by the World Health Organization (WHO) since the discovery of novel molecular markers in recent years [2]. A comprehensive molecular profiling of glioma can provide more detailed information on biological classifications.

The method of integrating histological and molecular characteristics heightens the objectivity of CNS tumor diagnosis and prompts the precision treatment for the CNS tumors. Mutations including IDH (*IDH1* and *IDH2*), 1p/19q codeletion, *EGFR* amplification, amplification of chromosome 7 and loss of chromosome 10 (+7/−10), *TERT* promoter alteration, *ATRX* deletion, *TP53* positive, and *CDKN2A/B* deletion are usually used for molecular diagnosis and stratification of most patients with gliomas [2]. Identification of such biomarkers in brain tumors is critical for the diagnosis, treatment, and prognosis of patients. The tumor tissue obtained from surgery has been widely used for clinical diagnosis and personalized medicine decision-making [3]. However, tumor tissue sampling in gliomas is affected by invasive craniotomy. The randomness of sampling means that it cannot completely represent the whole tumor feature. Liquid biopsy, a new cancer diagnostic technique, is minimally invasive and able to monitor potential dynamic changes in the tumors [4].

Cell-free circulating tumor DNA (ctDNA) is shed by tumor cells, and can be found in circulating blood, along with normal cells [5]. The clinical use of ctDNA has been evaluated in a variety of fields, including cancers [6]. Obviously, the amount of ctDNA in peripheral circulation is low by virtue of the low permeability of blood-brain barrier in gliomas [6, 7]. Cerebrospinal fluid (CSF) as an important source of potential molecular biomarkers contains various biomarkers, such as ctDNA, circulating tumor cells, miRNA, and extracellular vesicles, which are usually derived from brain tumor cells [8]. Previous studies showed that CSF was more sensitive than peripheral blood in the detection of DNA mutations, epigenetic alterations, and copy number variations, indicating the possibility of CSF ctDNA for precise diagnosis [9–11].

In this study, we performed a comprehensive genomic analysis based on targeted next-generation sequencing (NGS) of DNA from the tumor tissue and CSF ctDNA of 27 glioma patients, aiming at determining the feasibility of ctDNA from CSF in Chinese population. We compared the mutational and diagnostic concordance between the tumor tissue and CSF and revealed the utility of CSF ctDNA for advancing and complementing the diagnosis of gliomas.

## Materials and methods

### Patients and samples

We prospectively collected the clinical data of 27 glioma patients admitted to Nanjing Jinling Hospital (Nanjing, China) from December 2020 to July 2021. All patients were confirmed with glioma by magnetic resonance imaging (MRI). CSF samples (4–8 mL) were collected from the lumbar puncture before surgery. Tumor tissue samples were collected from surgical specimens, and part of removed tumor tissue was used for histopathologic diagnosis. 4 CSF samples were collected from patients with encephalitis as normal controls. In accordance with the Declaration of Helsinki, written informed consent was obtained from all individuals or guardians, and the study was approved by the Institutional Review Board of Nanjing Jinling Hospital (2022DZKY-093-01) and local regulations.

### DNA extraction and quantification

Cell-free DNA (cfDNA) was extracted using an Apostle MiniMax High Efficiency cfDNA Isolation Kit (APOSTLE) according to the manufacturer’s instructions. Fresh tissue DNA was extracted using a QIAamp DNA Tissue & Blood Kit (Qiagen) according to the manufacture’s recommendations. Extracted cfDNA and DNA were quantified by Qubit4.0 (Life Technologies) according to the manufacturer’s protocols. Quantitative levels of CSF cfDNA were measured in haploid genome equivalents per milliliter (hGE/mL), which was calculated as the result of total cfDNA concentration and the mean allele fraction of somatic mutations.

### NGS library establishment and sequencing

NGS libraries of tumor tissue DNA were prepared with KAPA HyperPlus Kit (Roche). cfDNA libraries were established with the xGenTM Prism DNA Library Prep Kit (IDT). After hybridization of DNA and cfDNA libraries, the captured libraries were amplified by polymerase chain reaction (PCR) according to the manufacturer’s protocols. As described previously, we performed the targeted sequencing by NGS with the panel containing 131 genes and 4 chromosomes (131 + 4 panel) related to brain tumors (see Supplementary Table S1 for gene list) [12]. Sequencing was carried out using NovaSeq 6000 system in this study.

### Bioinformatics analysis

All CSF and tumor samples were measured for single nucleotide variants (SNVs), copy number variants (CNVs), and chromosomal variations. Initial sequencing reads were trimmed and filtered with fastp (v.2.20.0) and then aligned against the human reference genome (hg19) using BWA-mem (v.0.7.17). The realignment of local regions was performed to improve the accuracy of variant detection around Indels using ABRA2.

We used VarDict (v.1.5.7) for variant detection. The results of variant detection were annotated to multiple databases using SnpEff and ANNOVAR software, including dbNSFP (v.4.2a), COSMIC (v.9.6), gnomAD (v.3.11), ClinVar and more. According to annotation results, the filtration was further conducted, and the background baseline was used to filter systematic false positive results. In combination of Intervar results and pathogenic interpretation from databases including ClinVar, somatic and germline mutations were finally identified. Threshold for mutation calling is ≥0.5% variant allele frequency (VAF) in CSF and ≥1.0% VAF in tumor tissue.

The original sequencing depth was counted, which was standardized to exclude the influence of the amount of sequencing data. The CNVkit algorithm was used to construct the background distribution model of the copy number with normal genomic sequencing. CNVs were determined based on the difference between the copy number in each region of the sample and the background model. The structural variations of chromosomes were determined according to the copy number distribution of the whole chromosome arm. CNVs were analyzed by CNVkit (dx1.1). We used CSF from the patients with non-tumor diseases as the baseline, and mutations that were identified as false positive were excluded.

### Immunohistochemical staining detection

Hematoxylin and eosin staining and immunohistochemistry (IHC) were performed on 4 µm-thick sections using standard protocols. IHC stains contains Ki-67 (Roche), and IDH1 R132H (Agilent).

### Integrated diagnosis

All cases were comprehensively diagnosed and reviewed by the two pathologists (NYL and NW) using histology, as well as molecular features of tumor DNA according to the 2021 WHO CNS classification criteria and the Consortium to Inform Molecular and Practical Approaches to CNS Tumour Taxonomy group in 2018–2020 [2, 13–16].

### Statistical analysis

Statistical analysis and plots were conducted by Graphpad Prism 5 and IBM SPSS statistics software version 19. Quantitative ctDNA differences across different characteristics of patients were assessed using Mann-Whitney U test. Spearman’s rank correlation coefficient test was used to evaluate the association between the tissue DNA and CSF ctDNA with SNVs and CNVs. *p*-values lower than 0.05 were considered as statistically significant. To assess the possibility of CSF ctDNA test for glioma diagnosis, we performed a receiver operating characteristic (ROC) curve analysis. Consistency of diagnosis was examined by Kappa measure of agreement. The Kappa values were categorized as “almost perfect” (0.8–1.0), “fair to good” (0.4–0.8), or “poor” (below 0.2) [17].

## Results

### Clinicopathological characteristics and detection of ctDNA levels

Twenty-seven gliomas were recruited for the CSF and tumor biopsy tissue collection. Histopathological subtypes included glioblastoma (GBM, *n* = 13), astrocytoma (*n* = 6), pilocytic astrocytoma (*n* = 2), pleomorphic xanthoastrocytoma (*n* = 2), oligodendroglioma (*n* = 1), ganglioglioma (*n* = 1), glioneuronal tumor (*n* = 1), and gliosarcoma (*n* = 1). The median age of the participants was 54 years (range 15–78 years) and 66.7% (*n* = 18) were male. These patients were confirmed pathologically and classified into histological grade I (*n* = 3), grade II (*n* = 6), grade III (*n* = 4), grade IV (*n* = 14). After surgery, 63.0% (*n* = 19) patients received standard Stupp treatments, including adjuvant radiotherapy and temozolomide (TMZ) chemotherapy, 11.1% (*n* = 3) patients received adjuvant radiotherapy. The characteristics of these patients were presented in Table 1.

**TABLE 1:** Patient characteristics, n(%).

Total	*n* = 27					
Age, years	54.1 ± 16.6					
Gender	Male			Female		
	18 (66.7)			9 (33.3)		
Histologic grade	I	II		III		IV
	3 (11.1)	6 (22.2)		4 (14.8)		14 (51.9)
Recurrence	1 (3.7)					
Post-Treatment	Treatment Naïve	RT		TMZ		TTField
	3 (11.1)	21 (77.9)		19 (70.4)		1 (3.7)
Tumor size	<57 mm		≥57 mm		Missing	
	15 (55.6)		9 (33.3)		3 (11.1)	
Tumor location	Supratentorial		Infratentorial		Missing	
	23 (85.2)		3 (11.1)		1 (3.7)	
MRI signal	Enhancement		Non-enhancement		Missing	
	15 (55.6)		8 (29.6)		4 (14.8)	
Characteristics	Multi lesions		Non-multi lesions		Missing	
	8 (29.6)		18 (66.7)		1 (3.7)	
	Involving ventricle		Not-involving ventricle		Missing	
	20 (74.1)		5 (18.5)		2 (7.4)	
	Involving cerebral cortex		Not-involving cerebral cortex		Missing	
	14 (51.9)		10 (37.0)		3 (11.1)	
	Ki 67 <20%		Ki 67 ≥20%		Missing	
	8 (29.6)		14 (51.9)		5 (18.5)	

Abbreviations: MRI, magnetic resonance imaging; RT, radiotherapy; TMZ, temozolomide; TTField, tumor-treating field.

**FIGURE 1:**
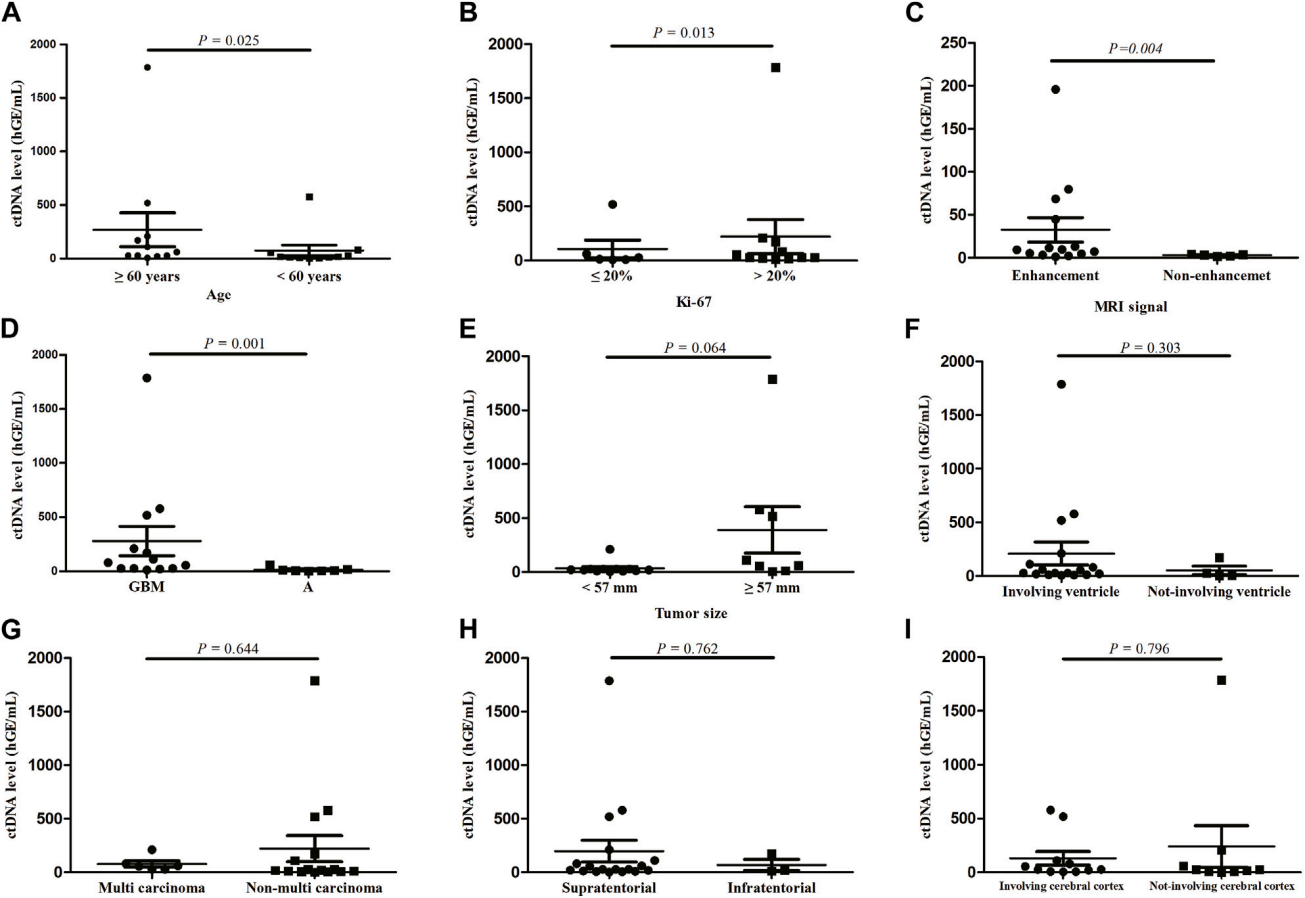
ctDNA levels according to patient characteristics, **(A)** age, **(B)** Ki-67, **(C)** MRI signal, **(D)** tumor subtypes, **(E)** tumor size, **(F)** ventricular involvement, **(G)** tumor number, **(H)** location, **(I)** involvement of cerebral cortex. Quantitative ctDNA levels were compared by Mann-Whitney U test. GBM, glioblastoma; A, astrocytoma.

We measured the ctDNA level among 27 CSF samples and found the ctDNA level showed significant differences across age (*p* = 0.025, Figure 1A), Ki-67 index (*p* = 0.013, Figure 1B), MRI signal (*p* = 0.004, Figure 1C), and tumor subtypes (*p* = 0.001, Figure 1D). There was no significant association of ctDNA levels with other clinical characteristics, including tumor size (*p* = 0.064), ventricular involvement (*p* = 0.303), tumor number (*p* = 0.644) and location (*p* = 0.762), as well as involvement of cerebral cortex (*p* = 0.769) (Figures 1E–I).

### Genomic profiling of gliomas from CSF ctDNA and tumor tissue DNA

**FIGURE 2:**
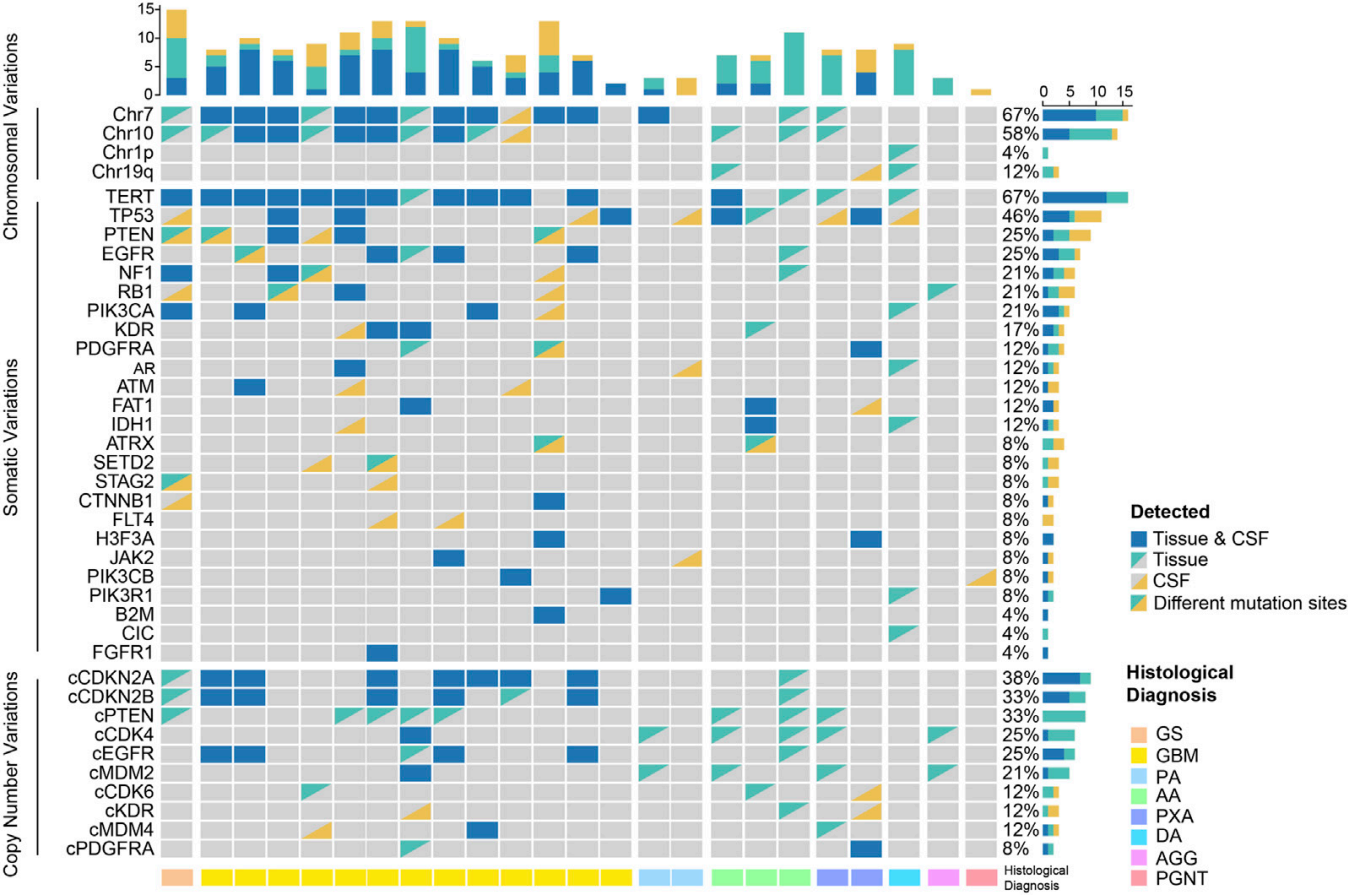
Genomic profiling in the spectra of CSF ctDNA and tumor DNA. GS, gliosarcoma; GBM, glioblastoma; PA, pilocytic astrocytoma; AA, anaplastic astrocytoma; PXA, pleomorphic xanthoastrocytoma; DA, diffuse astrocytoma; AGG, anaplastic ganglioglioma; PGNT, papillary glioneuronal tumor.

Twenty-seven patients were all detected using 131 + 4 panel, and 3 of them were excluded on the grounds of their undetectable ctDNA concentration. We identified at least 1 tumor-related gene mutation or chromosome variant from CSF ctDNA of 22 patients, consistent with the number of tumor tissue DNA samples (Figure 2). In contrast, we did not detect any oncogenic alterations in four individuals with encephalitis. Based on the cancer targeted panel, 32 mutant genes were detected in 24 gliomas. Of them, *TERT* was the most prevalent gene. *TERT* promoter mutations were detected in 12 (50%) of patients via CSF, while were identified in 16 (66.7%) of patients though tumor tissue. The concordance with the *TERT* mutation status between CSF and tumor tissue yielded 83.3%. The mutations were detected both in tumor tissue and CSF samples, apart from *FLT4* and *CIC* mutations. Abnormal gene copy number was found in 10 patients for CSF ctDNA. The coincidence between CSF and tumor tissue for *CDKN2A/B* deletion was 87.5%. However, 8 patients presented with *PTEN* deletion in tumor DNA but not detected in CSF ctDNA. Alterations of chromosome chr 1p, chr 19q, chr 7, or chr 10 were detected in 11 CSF samples.

### Concordance of SNVs and CNVs in matched tumor/CSF pairs

**FIGURE 3:**
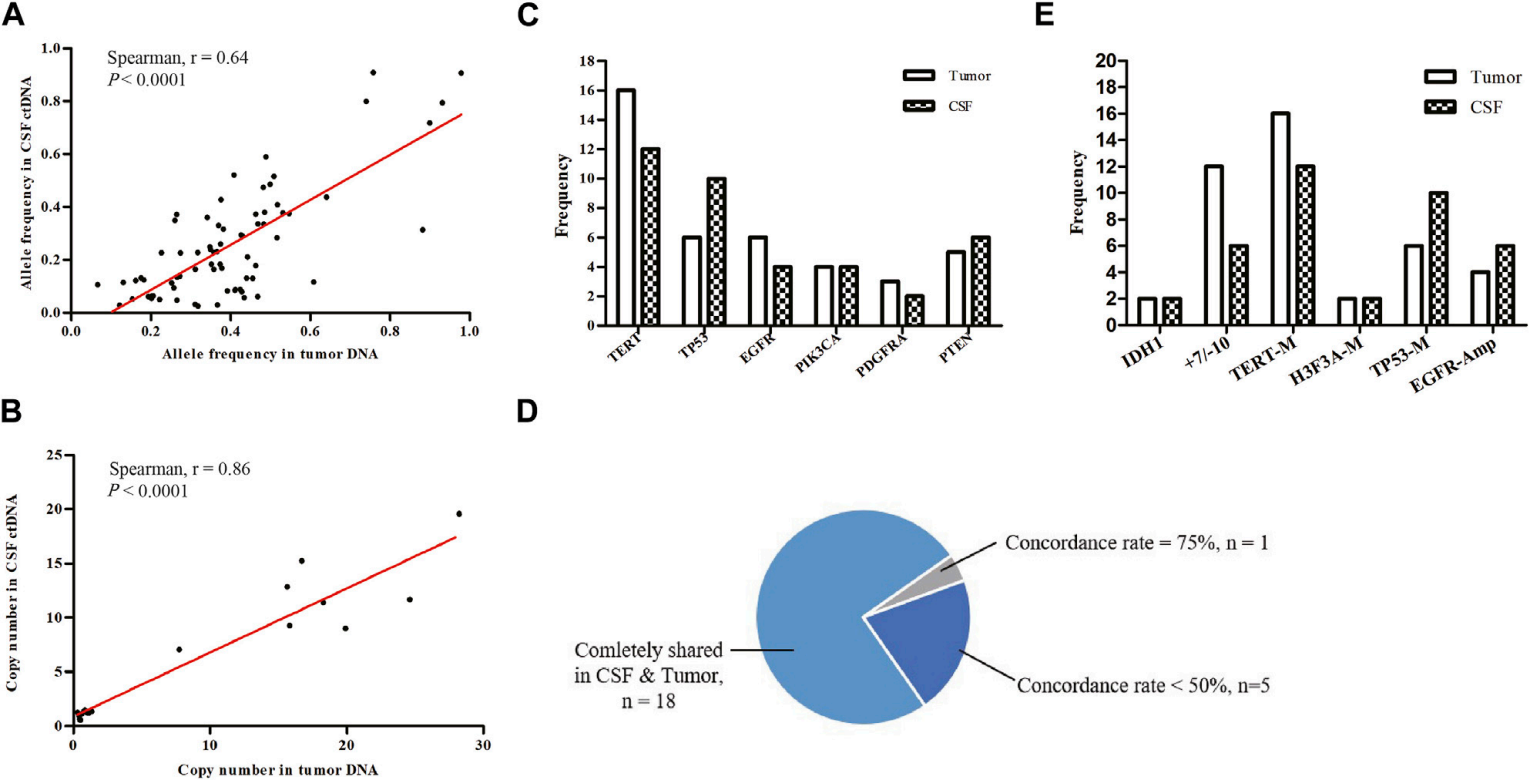
Mutational concordance between CSF ctDNA and tumor DNA. **(A)** Scatter plots of variant allele frequencies for all SNVs in CSF ctDNA and tumor DNA. **(B)** Scatter plots of copy number for all CNVs in CSF ctDNA and tumor DNA. Linear regression was calculated by Spearman correlation. Tumor and CSF profiles of high-frequency mutant genes **(C)** and key prognosis-related genes **(E)**. **(D)** Proportions of consistency between CSF and tumor high-frequency mutant genes were presented as a fan diagram.

We examined the concordance between SNVs and CNVs detected in tumor/CSF pairs. Comparison of CSF ctDNA and tumor DNA showed a moderately preferential correlation in the variant allele frequency (*r* = 0.64, *p* < 0.0001; Figure 3A) and a high correlation in the copy number (*r* = 0.86; *p* < 0.0001; Figure 3B). The most mutated genes in tumor samples and CSF ctDNA were *TERT*, *TP53*, *EGFR*, *PTEN*, *PIK3CA*, and *PDGFRA* (Figure 3C), which were completely shared in 18 patients, yielding an overall concordance rate of 81.8% between tumor and CSF ctDNA samples (Figure 3D). In addition, 5 out of 24 patients showed a gene concordance of less than 50%. Interestingly, the sensitivity of CSF in glioma was less than the tumor tissue in the detection of +7/−10 and *TERT* mutations, but presented a superior detection ability for *TP53* mutations and *EGFR* amplification (Figure 3E).

### Performance of CSF ctDNA in diagnosis of glioma

**FIGURE 4:**
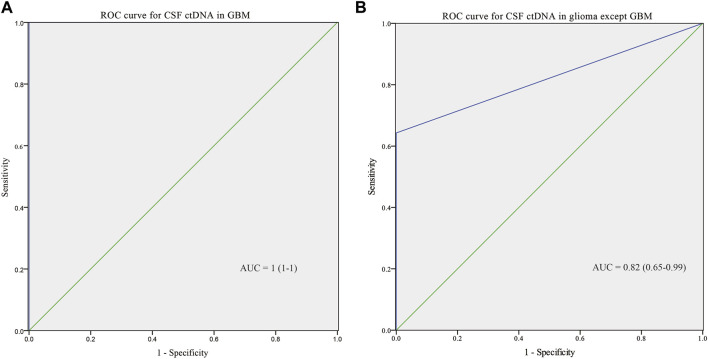
ROC curve for CSF ctDNA in GBM **(A)** and non-GBM **(B)**.

To further explore the performance of CSF ctDNA test, ROC curves for integrated diagnosis of all gliomas were generated (Figure 4). CSF ctDNA performed well in diagnosis of GBMs with the area under the curve (AUC) of 1 (95% CI: 1–1; Figure 4A). We also tested the possibility of CSF ctDNA as a strong classifier of non-GBM diagnosis (AUC = 0.82, 95% CI: 0.65–0.99, Figure 4B). When 3 cases with low ctDNA concentration was excluded, an AUC of 0.91 (95% CI: 0.77–1, Supplementary Figure S1A) was reached. Additionally, for patients with enhanced MRI signal, the AUC value was 1 (95% CI: 1–1; Supplementary Figure S1B).

**FIGURE 5:**
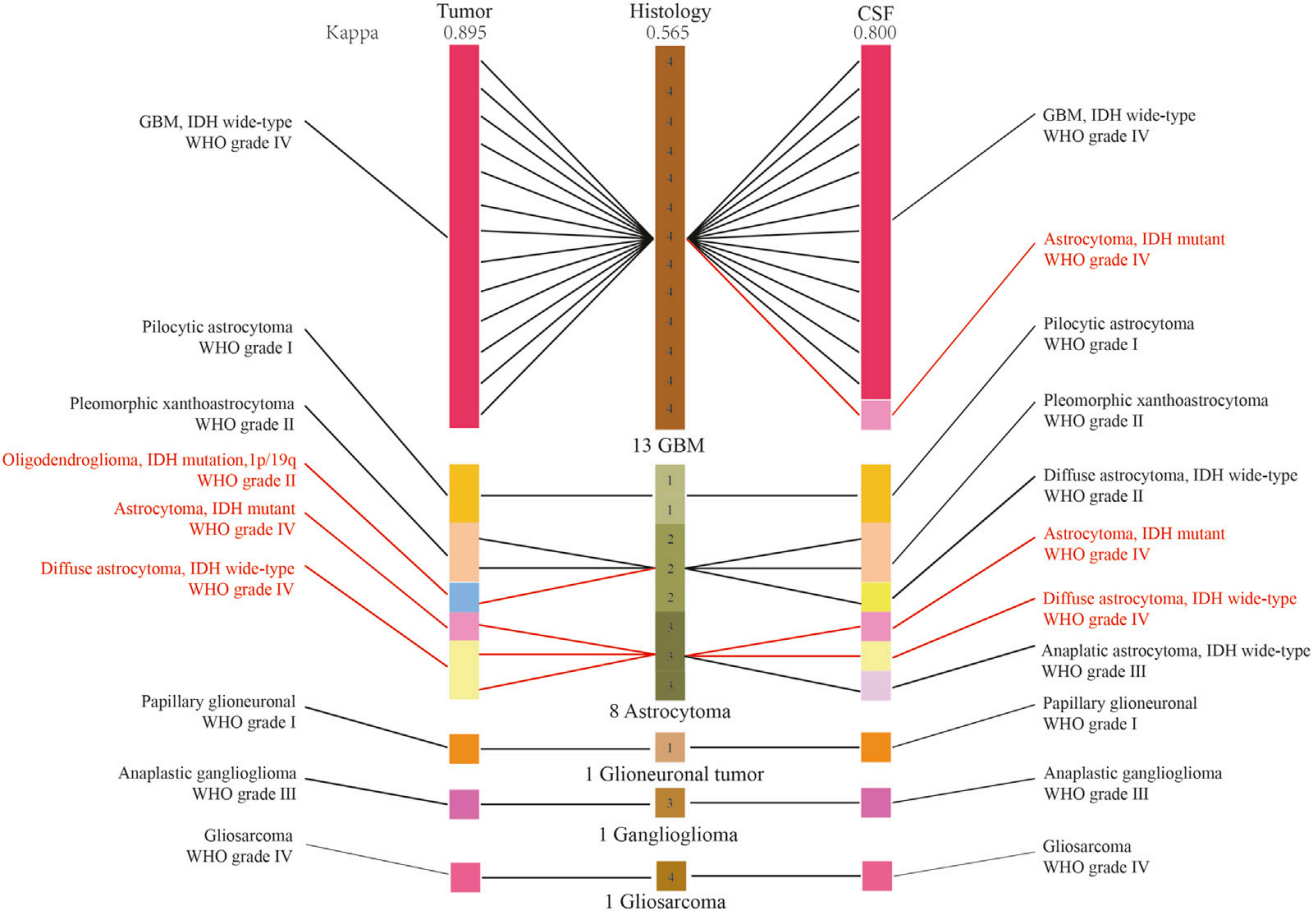
The diagnostic diagram of histology, and histology combined with CSF ctDNA and tumor DNA molecular features. The red character indicated the diagnoses were reclassified. Kappa measure of agreement was used to determine whether the diagnosis was consistent.

We established a new integrated diagnostic method by combining histology with tumor DNA and CFS ctDNA as the final diagnosis, which was used as the positive control. The diagnostic diagram of histology, and histology combined with CSF ctDNA and tumor DNA molecular features was revealed in Figure 5. The diagnosis of 5 patients were amended based on the integrated histological and molecular criteria, including 1 GBM and 4 astrocytomas. Among these, the diagnosis of 4 patients was rectified based upon histology and tumor DNA. In addition, there were 3 patients whose diagnosis was corrected depending on the histological and CSF ctDNA data. Kappa measure of agreement was used to determine whether the diagnosis was consistent and found the agreement between CSF and positive control was better. The agreement between CSF ctDNA and positive control was 91.6% (Kappa, 0.800), indicating a high concordance. Meanwhile, the conformity between histology and positive control was 79.1% (Kappa, 0.565), and the consistency in diagnosis between tumor DNA and positive control was 95.8% (Kappa, 0.895). The genotyping consistency of GBM was completely matched for CSF.

### Glioma subtyping and risk stratification based on CSF ctDNA

**FIGURE 6:**
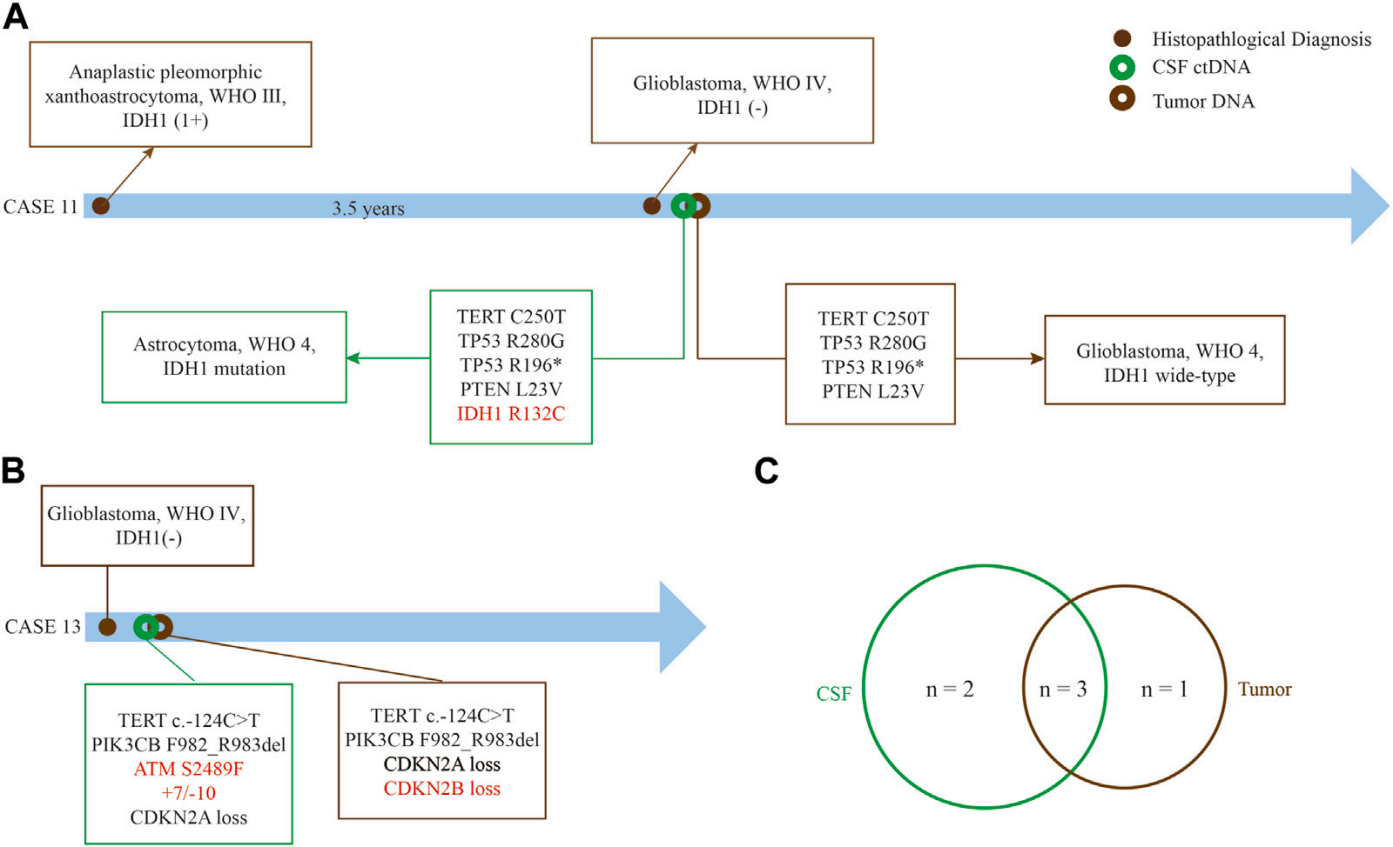
Superiority of CSF ctDNA detection in gliomas. **(A)** Case 11 who was diagnosed as recurrent GBM without *IDH1* mutation by tumor tissue but a rare IDH1 (R132C) mutation by CSF ctDNA sequencing. **(B)** Amplification of chromosome 7 and loss of chromosome 10 can be detected in case 13 by CSF ctDNA sequencing. **(C)** The common genes between CSF ctDNA and tumor tissue. The red font represents differential genes detected between CSF ctDNA and tumor tissue.

We found that CSF ctDNA sequencing could identify the glioma subtyping and determine the patient risk level. *IDH1/2* mutation was highly associated with the prolonged overall survival (OS).17 Case 11, a 59 year-old woman, was diagnosed as anaplastic pleomorphic xanthoastrocytoma, WHO Ⅲ, in September 2019, and recurred in March 2021 with histologically confirmed as GBM, WHO IV (Figure 6A). *IDH1* was positive at the time of initial diagnosis but negative at the time of recurrence according to immunohistochemical results. Next, tumor DNA sequencing failed to capture the mutation in *IDH1*, however, CSF ctDNA sequencing showed a rare mutation of R132C in IDH1, and corrected the tumor diagnosis into IDH1-mutated astrocytoma, WHO IV, according to CSF test. This patient experienced recurrence in the left temporal-occipital lobe, with greater possibilities of secondary GBM with IDH1 mutation. The remaining 4 genes were found as the shared alterations.

We also found different mutations between tumor DNA and CSF ctDNA sequencing. Case 13, a 78 year-old man with GBM, +7/−10 and *ATM* mutation were found in the CSF ctDNA but not in the tumor DNA, at the same time, loss of *CDKN2B* was only found in tumor DNA (Figure 6B). Three shared somatic mutation genes were identified between the tumor and the CSF, namely *TERT*, *PI3KCB*, and *CDKN2A* (Figure 6C). Importantly, these results suggested that the CSF ctDNA analysis can be invoked as an appropriate diagnostic tool to classify and stratify for some cases.

## Discussion

The 5 years survival rate of malignant CNS tumors was only 23.5%, which creates a great challenge for human health [1]. The publishing of the fourth and fifth edition of the WHO classification of tumors of the CNS underlined the importance of molecular diagnosis [2, 18]. Previous studies indicated that CSF could be used as an effective source of ctDNA for genotyping gliomas and monitoring tumor evolution [10, 19, 20], but the consistency of paired CSF and tumor of gliomas remains unclear. We prospectively collected 27 paired CSF and tumor tissue from surgical patients with gliomas and compared multiple gene mutations by using an NGS-based customized panel.

Currently, there are various methods for glioma liquid biopsy in CSF, such as Sanger sequencing, droplet digital (dd) PCR, and next-generation sequencing [21]. Sanger sequencing can detect gene variations with high precision. Since the ctDNA from CSF is limited, sequencing technologies should be improved [21]. Although ddRCR has satisfactory sensitivity for CSF ctDNA analysis, the throughput on variant analysis is still low and only can discover the known mutations [22]. NGS significantly compensates for Sanger sequencing and ddPCR deficiencies with improving the throughput of detection. Moreover, NGS can simultaneously capture known or unknown genetic mutations, cover the mutation types that cannot be detected by ddPCR or Sanger sequencing, and identify the chromosomal abnormalities [23]. Piccioni et al. found 55% of GBMs in blood ctDNA had at least one somatic alteration by a ctDNA NGS panel [24]. Miller et al. used NGS to analyze CSF samples and identified at least one tumor-derived DNA alteration in 42/85 patients with diffuse glioma [10]. We utilized a 131 + 4 gene panel based on NGS and demonstrated that 87.5% of gliomas harbored at least one somatic variation in CSF. Among the 24 patients with positive CSF cfDNA, we identified 18 cases with at least one mutation shared in tumor tissue and CSF. A prior study found that there was a shared somatic mutation between the tumor and CSF in pediatric, adolescent, and young adult brain tumors using MSK-IMPACT panel [20]. All CSF samples were obtained before surgery, aiming at aiding in integrated diagnosis. The sensitivity and specificity of our NGS-based method for CSF ctDNA detection was both better in GBM diagnosis, indicating that this detection technique may be appropriate for diagnosing higher-grade gliomas.

In our study, 3 patients (2 astrocytoma, 1 oligodendroglioma) with grade II diagnosed by histology showed ctDNA concentration below the detection limit. In general, people with CNS tumors harbored a very low ctDNA level in CSF, although it was much higher in CSF than in blood [6, 25]. Furthermore, the ctDNA level is also related to tumor stage. Lower-grade tumors usually have lower ctDNA levels, whereas higher-grade or metastatic tumors have higher levels [26, 27].

Tumor cell turnover is accelerated as tumor size and degree of malignancy, resulting in release of apoptotic and necrotic tumor cells into the circulation [26–28]. We discovered that patients with a high fraction of Ki-67 (>20%) had higher ctDNA levels, and that the ctDNA levels in GBM were much higher, but the difference in tumor size was not significant. Human Ki-67 expression was highly associated with the cell proliferation, indicating the association of Ki-67 index with the tumor progression [29]. GBM is the most malignant and aggressive glioma, accounting for 48.6% of all malignant CNS tumors [1]. Xu et al. considered that ctDNA for the diagnosis of advanced gastrointestinal stromal tumors was possible, and the Ki-67 affected the levels of ctDNA significantly [30]. We also found tumor DNA is not affected by the tumor location or number. The tumor DNA that invades the liquoral spaces can circulate throughout the entire CSF, causing to be collected from lumbar puncture in this study.

In our cohort, the hotspot aberrations detected involved *TERT* mutation, *TP53* mutation, *PTEN* mutation, *EGFR* mutation, and *CDKN2A/2B* loss. The molecular features were consistent with those of an adult glioma cohort previously reported [31]. *TERT* mutation is recognized as the most prevalent molecular marker for GBMs [32]. *H3F3A* G35R and K28M variants had been found in two gliomas separately, but no *IDH* mutations were detected in these two patients. It has been reported that mutations in *H3F3A* and *IDH1* were mutually exclusive [33]. The diagnostic accuracy rate of GBM by CSF ctDNA was higher compare of astrocytoma according to ROC analysis. A similar finding has also been described in a meta-analysis [34]. Generally, high-grade tumors contain more ctDNA to circulate. The concordance between diagnosis by CSF ctDNA and the final diagnosis was 91.6%. Compared with the tumor DNA, we found the existence of false negatives in some cases. The false negatives may be caused by the limited amount of ctDNA extracted, which limited the detection of variants with low allele frequency [35]. Low sensitivity in catching the variation of chromosome would limit the application of CSF ctDNA in low-grade gliomas.

CSF ctDNA detection exhibited some attractive features in help to observe the molecular biomarkers. We found a rare *IDH1* mutation in CSF ctDNA from case 11, whereas a negative result was found in tumor DNA. The samples we collected were prospectively obtained by removing a small section of the tumor. Intratumor heterogeneity may explain why sampling bias leads to a difference in biomarker discovery and not every driver or passenger mutation will be discovered in each cancer cell [36, 37]. CSF ctDNA can reflect the whole genetic heterogeneity and make longitudinal assessments possible [38]. IDH1/2 are enzymes that play critical roles in a variety of cellular functions such as carbohydrate metabolism, differentiation, DNA repair and redox state regulation [39, 40]. Arginine 132 is the hotspot of *IDH1* in gliomas and *IDH1* R132H mutations are the most predominant mutation which indicates a better prognosis and may be effective for inhibitor treatment [41]. In our study the *IDH1* mutation with R132C was found. Previous studies showed that R132C had a more favorable prognosis than R132H for gliomas with a much higher enzymatic metabolism efficiency [40, 42]. *EGFR* amplification, +7/−10, and *TERT* promoter mutation are alterations frequently observed in adult *IDH*-wildtype GBM [43]. Notably, a case of GBM was identified to harbor +7/−10 and *TERT* promoter mutations in CSF ctDNA. Weller *et al.* found *TERT* promoter mutations combined with +7/−10 had the worst progression-free survival and OS compared with those having TERT promoter mutation alone [44]. Importantly, CSF can be collected before tumor tissue, which means earlier diagnosis and timely treatment for the patients.

Precise tumor subtyping and grading are essential for prognosis and treatment of patients. Our present study demonstrated that CSF ctDNA may be a reliable alternate tumor source for integrated molecular diagnosis of gliomas, especially for GBMs, supported by previous studies [45, 46]. In lower-grade gliomas, both CSF and tumors showed unique mutant genes, indicating that CSF may act as a potential supplementary tool for tumor biopsy to improve the molecular diagnosis. Collectively, this novel integrated diagnosis based on histology, tumor DNA and CSF ctDNA molecular pathology revealed a powerful clinical diagnostic value. Due to the limitations of CSF sources, we did not conduct any further validation tests. To further confirm the utilization of CSF, larger cohorts and validation testing should be used.

In conclusion, CSF ctDNA detection for integrated molecular diagnosis of gliomas especially GBMs is feasible. NGS-based detection of CSF ctDNA could be used as a supplement for integrated diagnosis of gliomas. In the future, CSF ctDNA sequencing will form genetic profiling for each patient, assisting in targeted therapy and enhancing the therapeutic effect.

## Data Availability

The raw data supporting the conclusion of this article will be made available by the authors, without undue reservation.

## Supplementary material

SUPPLEMENTARY FIGURE S1 ROC curve for CSF ctDNA in non-GBM. **(A)** and glioma with enhanced MRI signal. **(B)** mergeindicator:supplemental_dummy:start.
