# Supplementary material for: Concordance analysis of cerebrospinal fluid with the tumor tissue for integrated diagnosis in gliomas based on next-generation sequencing
Source: Pathol Oncol Res. 2023 Sep 26;29:1611391. doi: 10.3389/pore.2023.1611391 (PMC10562547; doi:10.3389/pore.2023.1611391)
Supplement: Supplementary file 1 [file Table1.DOCX]

**Supplemental Table 1.** Genes and chromosomes covered by 131+4 gene panel.

| Chromosomes and gene names | | | | | | | | |
| --- | --- | --- | --- | --- | --- | --- | --- | --- |
| *ACVR1* | *AKT1* | *AKT2* | *ALK^*&^* | *APC* | *AR* | *ARAF* | *ARID2* | *ATM* |
| *ATRX* | *B2M* | *BCL2L11^*^* | *BCOR* | *BRAF*^&^* | *BRCA1* | *BRCA2* | *CBL* | *CCND2* |
| *CDK4* | *CDK6* | *CDKN2A* | *CDKN2B* | *CDKN2C* | *CHEK2* | *CIC* | *CTNNB1* | *DAXX* |
| *DDR2* | *DDX3X* | *DICER* | *DNMT3A* | *EGFR* | *EPCAM^#^* | *ERBB2* | *ERBB3* | *ERBB4* |
| *ESR1* | *FAT1* | *FBXW7* | *FGF4* | *FGFR1* | *FGFR2* | *FGFR3^*&^* | *FGFR4* | *FLT3* |
| *FLT4* | *FUBP1* | *GNA11* | *GNAQ* | *GNAS* | *H3F3A* | *HDAC4* | *HIST1H3B* | *HIST1H3C* |
| *HMCN1* | *HNF1A* | *HRAS* | *IDH1* | *IDH2* | *IRS2* | *JAK1* | *JAK2* | *KDM5A* |
| *KIT* | *KLF4* | *KRAS* | *MAP2K1* | *MAPK1* | *MDM2* | *MDM4* | *MEN1* | *MET^*&#^* |
| *MLH1* | *MPL* | *MSH2* | *MSH6* | *MTOR* | *MYB^*&^* | *MYC* | *MYCN* | *NAB2^*&^* |
| *NF1* | *NF2* | *NOTCH1* | *NR3C1* | *NRAS* | *NTRK1^*&^* | *NTRK2*^&^* | *NTRK3^*&^* | *PDGFRA* |
| *PDGFRB* | *PIK3CA* | *PIK3CB* | *PIK3R1* | *PLCG1* | *PMS2* | *POLE* | *POLR2A* | *PPM1D* |
| *PTCH1* | *PTEN* | *PTPN11* | *RAF1* | *RB1* | *RELA^*&^* | *RET^*&^* | *RGPD3* | *RICTOR* |
| *ROS1^*&^* | *SDHA* | *SETD2* | *SMAD4* | *SMARCA4* | *SMARCB1* | *SMARCE1* | *SMO* | *SRC* |
| *STAG2* | *STAT6^*&^* | *TERT^#^* | *TP53* | *TRAF7* | *TSC1* | *TSC2* | *USP8* | *VEGFA* |
| *VEGFB* | *VEGFR1* | *VEGFR2* | *VHL* | *YAP1^*&^* | chr 1p | chr 19q | chr +7 | chr -10 |

**Notes:** The SNVs, Indels and CNVs of all genes were tested. Chr: chromosome structural variants; &: gene fusions; *: containing the intron zone; #: involving the promoter region.
