# Supplementary figures and images for: Concordance analysis of cerebrospinal fluid with the tumor tissue for integrated diagnosis in gliomas based on next-generation sequencing
Source: Pathol Oncol Res. 2023 Sep 26;29:1611391. doi: 10.3389/pore.2023.1611391 (PMC10562547; doi:10.3389/pore.2023.1611391)

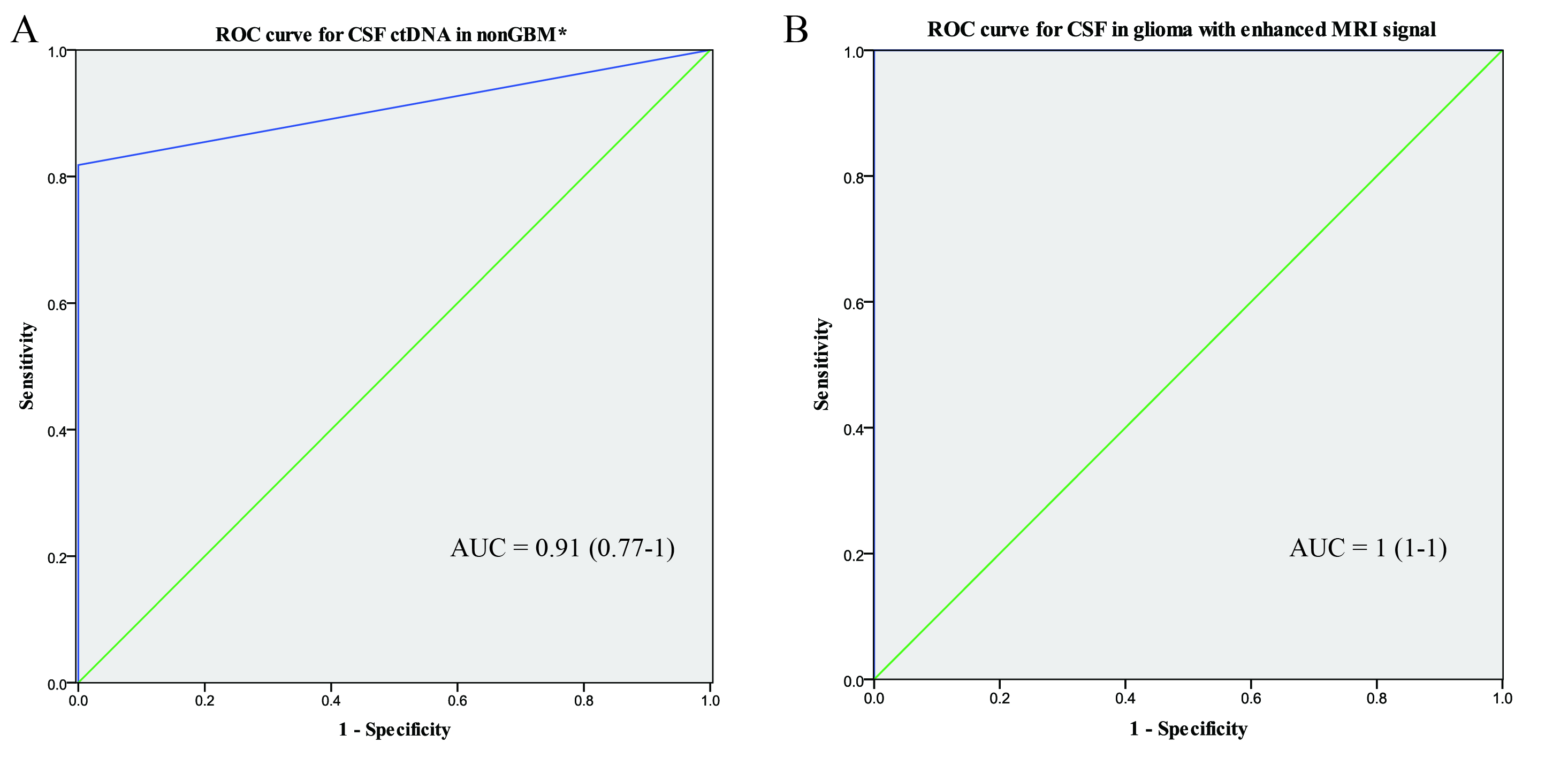

Supplement: Supplementary file 2 [file Image1.JPEG]
